# Eco-epidemiological assessment of the COVID-19 epidemic in China, January–February 2020

**DOI:** 10.1080/16549716.2020.1760490

**Published:** 2020-05-14

**Authors:** Peter Byass

**Affiliations:** Department of Epidemiology and Global Health, Umeå University, Umeå, Sweden; Aberdeen Centre for Health Data Science (ACHDS), Institute of Applied Health Sciences, School of Medicine, Medical Sciences and Nutrition, University of Aberdeen, Aberdeen, Scotland; MRC/Wits Rural Public Health and Health Transitions Research Unit (Agincourt), School of Public Health, Faculty of Health Sciences, University of the Witwatersrand, Johannesburg, South Africa

**Keywords:** COVID19, SARS-CoV-2, corona virus, weather, China

## Abstract

**Background**: The outbreak of COVID-19 in China in early 2020 provides a rich data source for exploring the ecological determinants of this new infection, which may be of relevance as the pandemic develops.

**Objectives**: Assessing the spread of the COVID-19 across China, in relation to associations between cases and ecological factors including population density, temperature, solar radiation and precipitation.

**Methods**: Open-access COVID-19 case data include 18,069 geo-located cases in China during January and February 2020, which were mapped onto a 0.25° latitude/longitude grid together with population and weather data (temperature, solar radiation and precipitation). Of 15,539 grid cells, 559 (3.6%) contained at least one case, and these were used to construct a Poisson regression model of cell-weeks. Weather parameters were taken for the preceding week given the established 5–7 day incubation period for COVID-19. The dependent variable in the Poisson model was incident cases per cell-week and exposure was cell population, allowing for clustering of cells over weeks, to give incidence rate ratios.

**Results**: The overall COVID-19 incidence rate in cells with confirmed cases was 0.12 per 1,000. There was a single confirmed case in 113/559 (20.2%) of cells, while two grid cells recorded over 1,000 confirmed cases. Weekly means of maximum daily temperature varied from −28.0°C to 30.1°C, minimum daily temperature from −42.4°C to 23.0°C, maximum solar radiation from 0.04 to 2.74 MJm^−2^ and total precipitation from 0 to 72.6 mm. Adjusted incidence rate ratios suggested brighter, warmer and drier conditions were associated with lower incidence.

**Conclusion**: Though not demonstrating cause and effect, there were appreciable associations between weather and COVID-19 incidence during the epidemic in China. This does not mean the pandemic will go away with summer weather but demonstrates the importance of using weather conditions in understanding and forecasting the spread of COVID-19.

## Background

In infectious outbreak situations, much epidemiological effort rightly goes into case-finding and follow-up in order to track epidemics. Population-based analyses of ecological factors are however also important, not least to inform models and prognostics for future spread of the same infection elsewhere. Where a new disease is involved, such as COVID-19, it is particularly important to chart an unknown infectious agent’s interactions with environments in which transmission has occurred.

The large-scale outbreak of COVID-19 in China at the start of 2020, by now largely contained, presents an important opportunity to carry out an eco-epidemiological assessment which may be relevant for understanding patterns of transmission relevant for the ensuing pandemic. Unprecedented open-access data at the individual case level for the outbreak in China, including geo-location data, plus the availability of detailed remote-sensed and global-gridded ecological data, make this possible. The ecological dimensions of the pathogen–host relationship are likely to be determined by multiple factors, including population density, social contacts, a range of environmental and meteorological parameters, and population-based control measures. This assessment specifically looks at weather and COVID-19 incidence on a small-area basis in China, adjusting for population density and week of the epidemic.

Many well-established pathogens follow well-known seasonally and ecologically determined patterns of activity [1]. The established concept of ‘tropical medicine’ was largely predicated around pathogens and vectors predominantly localised in tropical regions, with some cases in travellers manifesting elsewhere [2]. Obviously little is yet known about seasonal and ecological patterns for the new SARS-CoV-2 coronavirus causing the current COVID-19 pandemic, although it is already clear that this pathogen has capacity for wide and rapid geographic spread, and it is not confined to any particular climatic zone. Nevertheless, SARS-CoV-2 transmission may still be mediated by local weather conditions. Other coronaviruses, such as Middle East Respiratory Syndrome Coronavirus (MERS-CoV) have been shown to follow established seasonal patterns [3]. Human coronavirus infections have been found to be more common in winter in Norway [4], and in Israel in summer [5]. Thus, other coronavirus diseases show various weather-related transmission patterns, which may also be true for COVID-19.

The original COVID-19 epicentre in Wuhan City reported approximately 3 times as many confirmed cases as the whole of the rest of China, with peak incidence during January 2020 [6]. This overwhelming number of cases in a single location was not included in analyses here since it did not contribute to geographic variation and occurred slightly earlier than the generalised epidemic in China. While the actual number of COVID-19 cases in China is generally considered to be much higher than the number of tested and confirmed cases, perhaps by as much as 20-fold [7], confirmed cases represent a reasonable basis for deriving incidence rate ratios in relation to environmental factors. It is unlikely in general that weather conditions would systematically affect the ratio of confirmed:unconfirmed cases.

Several other recent studies have addressed the relationship between weather and COVID-19 transmission. One study looked at average temperature and relative humidity, but only measured at a provincial level, did not consider solar radiation and rainfall, and did not reach clear public health conclusions [8]. Another study related reproductive numbers in Chinese cities to temperature and humidity and extrapolated those findings worldwide [9]. Large-scale modelling of temperature and humidity in relation to COVID cases suggested associations [10]. Some attempts to model global transmission with meteorological parameters have been made [11]. A National Academies rapid review concentrated primarily on limited laboratory studies of various exposures to the SARS-CoV-2 virus, and also concluded that available natural history studies (not including this one) were so far inconclusive [12].

This eco-epidemiological assessment set out to use data on COVID-19 incident cases throughout China during January and February 2020 (excluding the original epidemic focus of Wuhan city) and to relate them to week of confirmation, population and meteorological data. This represents a kind of ‘natural experiment’ in terms of how secondary epidemic foci occurred in diverse locations around China, before the national epidemic was more or less under control by the end of February, with wide variation in week-specific and location-specific incidence rates. The objective was to characterise the extent to which ecological factors may have mediated COVID-19 transmission in China to inform public health planning and modelling in other settings.

## Methods

This assessment is based on the open-access COVID-19 incident case data maintained by the Open COVID-19 Data Curation Group [13]. A total of 18,069 geo-located COVID-19 incident confirmed cases during January and February 2020 were extracted for the whole of China, excluding Wuhan City. No confirmed cases were reported for the first 2 weeks of January. All cases were mapped by week of confirmation onto a 0.25° latitude/longitude grid (approximately 25 × 25 km squares) for the whole of China, which included 15,539 cells. This grid was also filled with population data from the NASA Socioeconomic Data and Applications Center [14]. Gridded weather data for the whole of China during January and February 2020 were sourced from the Copernicus Climate Change Service ERA-5 T model, specifically temperature at 2 m, total precipitation and total sky direct solar radiation at surface [15]. Weekly averages/totals of daily weather data for each geographic cell were calculated and mapped on the geographic grid. These data were used as the basis for generating maps of cases and ecological factors.

There were 559/15,539 (3.6%) grid cells which contained at least one reported case at some point during January and February (excluding Wuhan City), and these were used to construct a Poisson regression model (Stata 12) in which the unit of observation was cell-week, over the period during which cases occurred anywhere in the country (weeks 3 to 9 of 2020, total 3,913 units of observation). Weather parameters during the preceding week were included in the model on the basis of the established 5–7 day incubation period for COVID-19, since this was likely to reflect weather at the time of disease transmission [16]. The dependent variable in the Poisson model was the number of incident confirmed cases in the cell-week and exposure was the population in the cell, allowing for clustering by the grid cell identifier over weeks, with incidence rate ratio as the outcome. In the absence of established hypotheses on relationships between ecological factors and SARS-CoV-2 transmission, tertiles of quantitative variables were constructed as independent variables to avoid erroneously imposing linear assumptions. Grid cells which had no incident cases reported throughout January and February were excluded from the regression model, in the absence of any evidence of the possibility of transmission in the cell.

## Results

A total of 18,069 confirmed cases were reported, contained in 559 0.25° grid cells, which corresponded to an overall population of 151.2 million, around 10% of the total Chinese population. This amounted to an overall COVID-19 confirmed case incidence rate in cells with cases of 0.12 per 1,000 population, although the incidence of confirmed cases should be taken to represent a small proportion of all cases. In 113/559 (20.2%) of grid cells with cases, there was only one confirmed case during the whole period, while two grid cells recorded over 1,000 cases.

**Figure 1.**
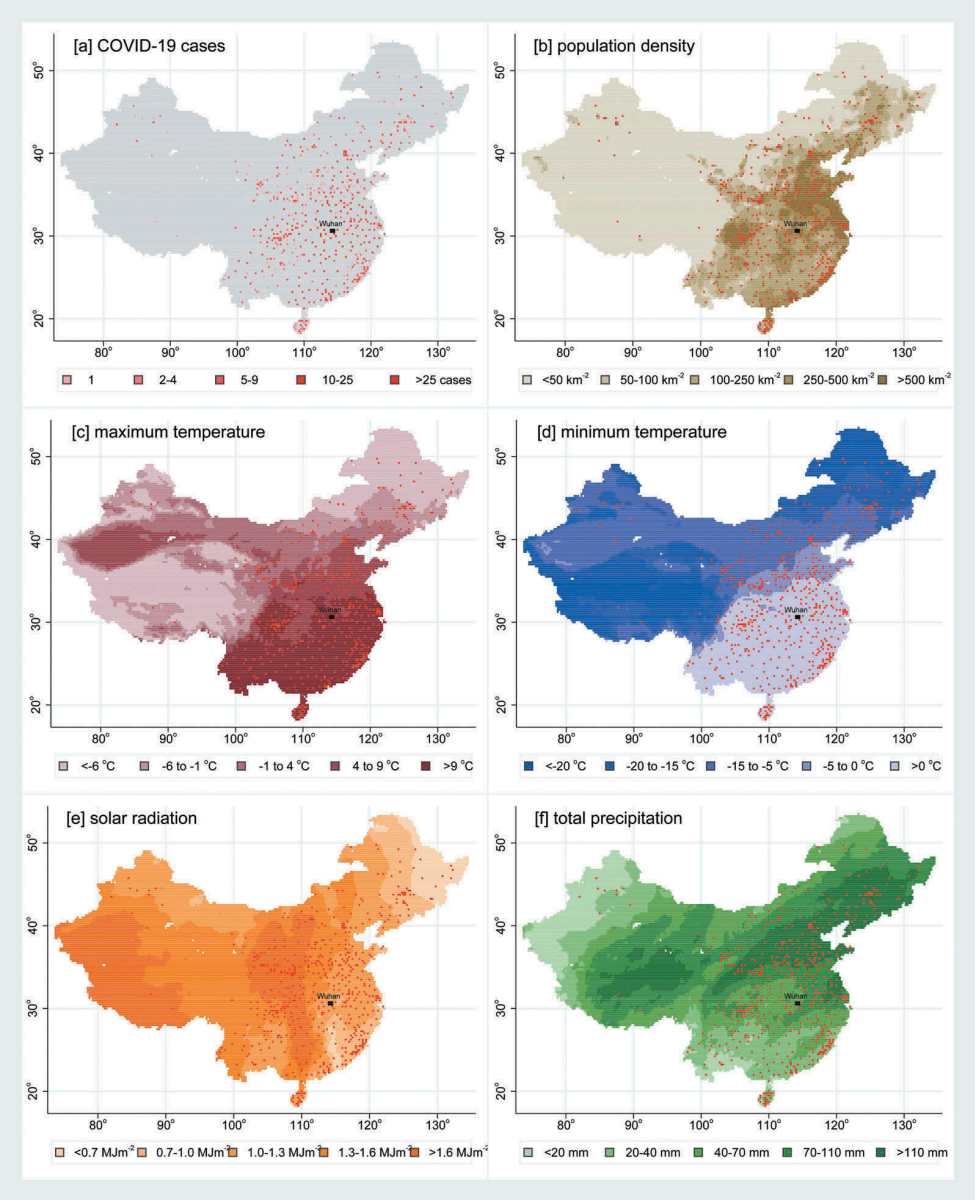
Maps of China, based on 15,539 0.25° grid cells, showing, for January–February 2020, (a) cell-densities of COVID-19 cases, (b) population density, (c) maximum temperature, (d) minimum temperature, (e) solar radiation and (f) precipitation. Maps (b–f) show the 559 cells having at least one case in red (these maps are approximate representations of national borders)

Figure 1 shows (a) the geographic distribution of cases, (b) population density for the whole country, and, for January–February 2020, (c) average of daily maximum temperature at 2 m, (d) average of daily minimum temperature at 2 m, (e) total precipitation and (f) average of daily total sky direct solar radiation at the surface. The Supplementary Material contains similar maps for temperature, precipitation and solar radiation on a weekly basis, showing the location of cases confirmed in the following week. Weekly means of maximum daily temperature varied from −28.0°C to 30.1°C, minimum daily temperature from −42.4°C to 23.0°C, maximum solar radiation from 0.04 to 2.74 MJm^−2^ and total precipitation from 0 to 72.6 mm.

**Figure 2.**
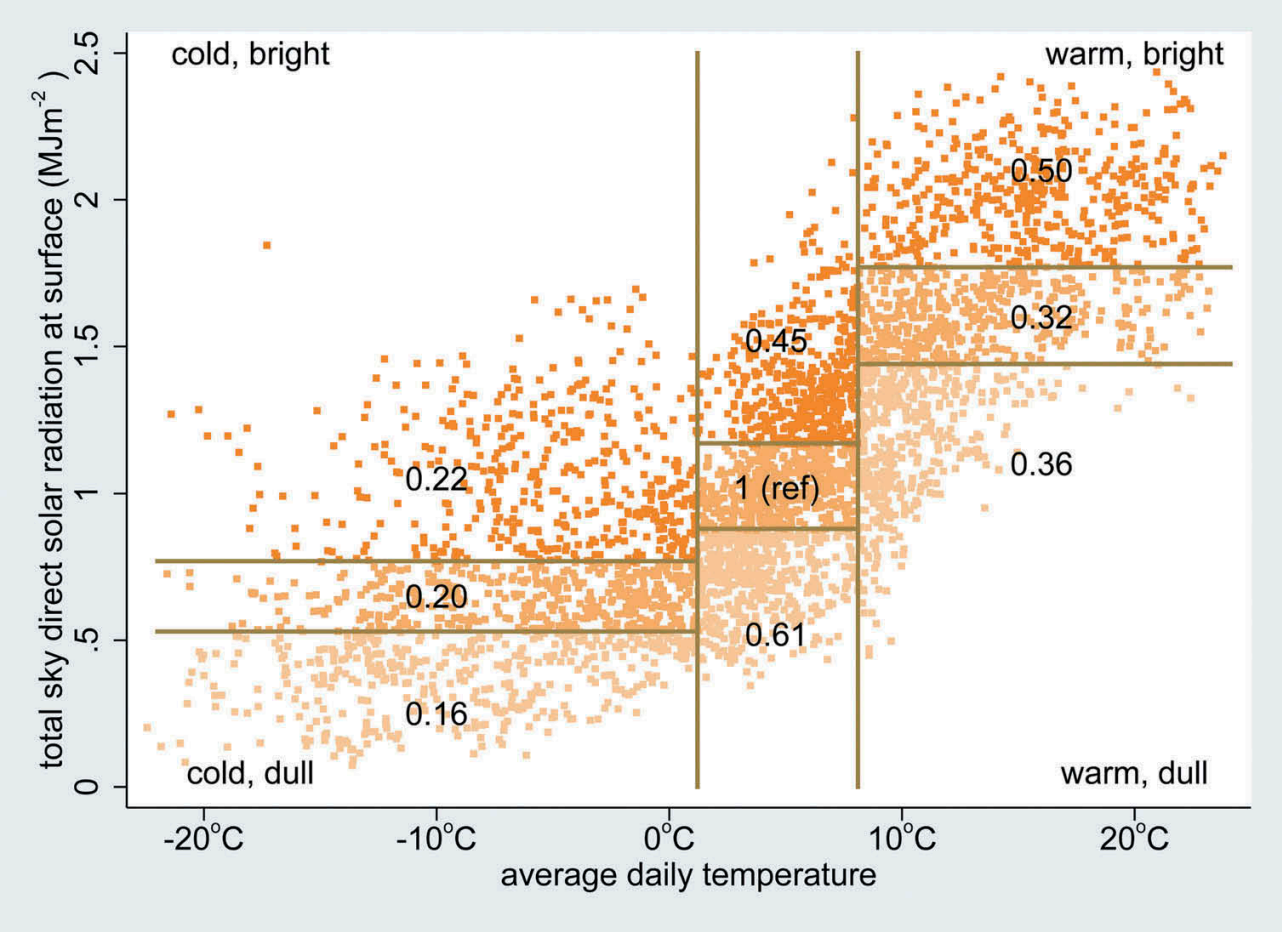
Weekly means of average daily temperature and solar radiation for 3,913 0.25° grid cell-weeks (shown as dots) of observation for COVID-19 incident cases in China (excluding Wuhan City) during January–February 2020, covering a total of 18,069 cases among a population of 151.2 million. Temperature is divided into tertiles, with each tertile then divided into tertiles of solar radiation. Numbers in each sector represent COVID-19 incidence rate ratios (adjusted for week, population density and precipitation)

Table 1 summarises the data included in building a Poisson regression model. Because of concerns about collinearity between maximum and minimum temperatures and solar radiation for constructing a multivariable regression model, average daily temperature was calculated as the average of maximum and minimum daily temperatures, as a single measure of temperature. Then, to avoid imposing linear relationships between temperature, solar radiation and COVID-19 incidence, which appeared improbable from the bivariable results in Table 1, a tertiles of tertiles approach was used, in which tertiles of average temperature were further broken down into tertiles of solar radiation, as shown in Figure 2. The nine categories derived in this way were then used as a categorical independent variable in the regression model, rather than having separate competing variables for temperature and solar radiation.

**Table 1.** Details of 3,913 0.25° grid cell-weeks of observation for COVID-19 incident cases in China (excluding Wuhan City) during January–February 2020, covering a total of 18,069 cases among a population of 151.2 million. All grid cells in which at least one case was observed during the overall period of observation are included. Bivariate incidence rate ratios and their 95% confidence intervals were calculated using a Poisson regression model with the weekly number of cases as the dependent variable, the grid cell population as the exposure variable and the previous week’s weather data as independent variables

Parameter	Level	Units	Range	Median	Bivariate incidence rate ratio	95% confidence interval
Latitude		degrees	18.25 to 49.75	32.25		
Longitude		degrees	82.00 to 132.25	113.25		
Population	per grid cell	n	1,169 to 2.56 million	187,616		
COVID-19 cases	per grid cell	n	1 to 1,262	6		
Week in 2020	3				1 (ref)	–
	4				27.5	22.7–33.4
	5				84.9	70.2–102.7
	6				38.9	32.1–47.0
	7				11.8	9.7–14.4
	8				1.80	1.4–2.3
	9				1.38	1.1–1.8
Population density	1st quintile	km^−2^	2 to 109	57	1 (ref)	–
	2nd quintile	km^−2^	110 to 198	155	0.75	0.69–0.81
	3rd quintile	km^−2^	199 to 392	275	1.01	0.94–1.08
	4th quintile	km^−2^	393 to 631	489	0.67	0.63–0.72
	5th quintile	km^−2^	632 to 3,626	788	0.20	0.18–0.21
Preceding week mean of daily maximum temperature	1st tertile	°C	−18.1 to 5.2	−0.3	1 (ref)	–
	2nd tertile	°C	5.2 to 11.7	8.5	2.11	2.02–2.20
	3rd tertile	°C	11.7 to 29.2	16.1	1.00	0.96–1.05
Preceding week mean of daily minimum temperature	1st tertile	°C	−31.2 to −3.1	−11.3	1 (ref)	–
	2nd tertile	°C	−3.1 to 4.5	1.4	3.27	3.11–3.42
	3rd tertile	°C	4.5 to 15.9	8.3	1.13	1.07–1.19
Preceding week mean of daily average temperature	1st tertile	°C	−22.4 to 1.2	−5.7	1 (ref)	–
	2nd tertile	°C	1.2 to 8.1	4.8	3.58	3.42–3.76
	3rd tertile	°C	8.1 to 19.4	12.0	1.11	1.05–1.17
Preceding week mean of daily max solar surface radiation	1st tertile	MJm^−2^	0.07 to 0.82	0.59	1 (ref)	–
	2nd tertile	MJm^−2^	0.59 to 1.33	1.07	2.57	2.48–2.67
	3rd tertile	MJm^−2^	1.33 to 2.44	1.64	0.72	0.69–0.76
Preceding week total precipitation	1st tertile	mm	0 to 4.1	1.8	1 (ref)	–
	2nd tertile	mm	4.1 to 11.4	7.2	2.57	2.44–2.70
	3rd tertile	mm	11.4 to 55.9	17.2	4.71	4.50–4.94

If the single confirmed cases recorded in 113 cells genuinely represented local index cases who had travelled from elsewhere in China after becoming infected under different ecological conditions, it could be argued that they might distort the results. Though it was probably more likely that the single confirmed cases were contacts of local index cases, a sensitivity analysis was performed by repeating the regression with the omission of the 113 cells with single confirmed cases. The results of the regression model and this sensitivity analysis are shown in Table 2, with no meaningful differences seen after excluding the cells with single confirmed cases.

**Table 2.** Details of 3,913 0.25° grid cell-weeks of observation for COVID-19 incident cases in China (excluding Wuhan City) during January–February 2020, covering a total of 18,069 cases among a population of 151.2 million. Results are shown separately for all 3,913 cell-weeks and with the exclusion of single-case cells as a sensitivity analysis. Multivariable incidence rate ratios and their 95% confidence intervals were calculated using a Poisson regression model with the weekly number of cases as the dependent variable, the grid cell population as the exposure variable and the previous week’s weather data as independent variables

Parameter	Level	All 3,913 cell-weeks		Sensitivity analysis excluding single-case cells	
		Multivariable incidence rate ratio	95% confidence interval	Multivariable incidence rate ratio	95% confidence interval
Week in 2020	3	1 (ref)	–	1 (ref)	–
	4	19.4	11.1–33.8	19.7	11.3–34.2
	5	48.0	16.7–86.7	50.8	28.5–91.5
	6	21.8	11.7–40.7	24.6	13.4–45.1
	7	10.3	5.92–17.8	11.4	6.66–19.6
	8	1.39	0.67–2.88	1.55	0.75–3.22
	9	0.90	0.34–2.33	1.02	0.39–2.65
Population density quintiles	9–109 km^−2^	1 (ref)	–	1 (ref)	–
	111–198 km^−2^	0.52	0.25–1.12	0.54	0.25–1.16
	199–392 km^−2^	0.57	0.29–1.13	0.53	0.27–1.04
	393–631 km^−2^	0.29	0.14–0.61	0.30	0.14–0.63
	632–3,626 km^−2^	0.11	0.05–0.20	0.10	0.05–0.19
Preceding week temperature and solar radiation tertiles of tertiles	1.2–8.1°C; 0.88–1.17 MJm^−2^	1 (ref)	–	1 (ref)	–
	1.2–8.1°C; 0.35–0.88 MJm^−2^	0.61	0.36–1.05	0.66	0.40–1.09
	1.2–8.1°C; 1.17–2.28 MJm^−2^	0.45	0.30–0.69	0.43	0.29–0.66
	8.1–23.8°C; 1.44–1.77 MJm^−2^	0.32	017–0.62	0.30	0.16–0.58
	8.1–23.8°C; 0.44–1.44 MJm^−2^	0.36	0.20–0.64	0.34	0.19–0.60
	8.1–23.8°C; 1.77–2.44 MJm^−2^	0.50	0.26–0.95	0.44	0.23–0.84
	−22.4–1.2°C; 0.53–0.77 MJm^−2^	0.20	0.10–0.41	0.24	0.12–0.47
	−22.4–1.2°C; 0.07–0.53 MJm^−2^	0.16	0.09–0.27	0.18	0.11–0.29
	−22.4–1.2°C; 0.77–1.84 MJm^−2^	0.22	0.11–0.43	0.24	0.05–0.19
Preceding week total precipitation tertiles	0–4.1 mm	1 (ref)	–	1 (ref)	–
	4.1–11.4 mm	1.50	1.11–2.03	1.45	1.08–1.95
	11.4–55.9 mm	2.12	1.56–2.88	1.97	1.46–2.65

**Figure 3.**
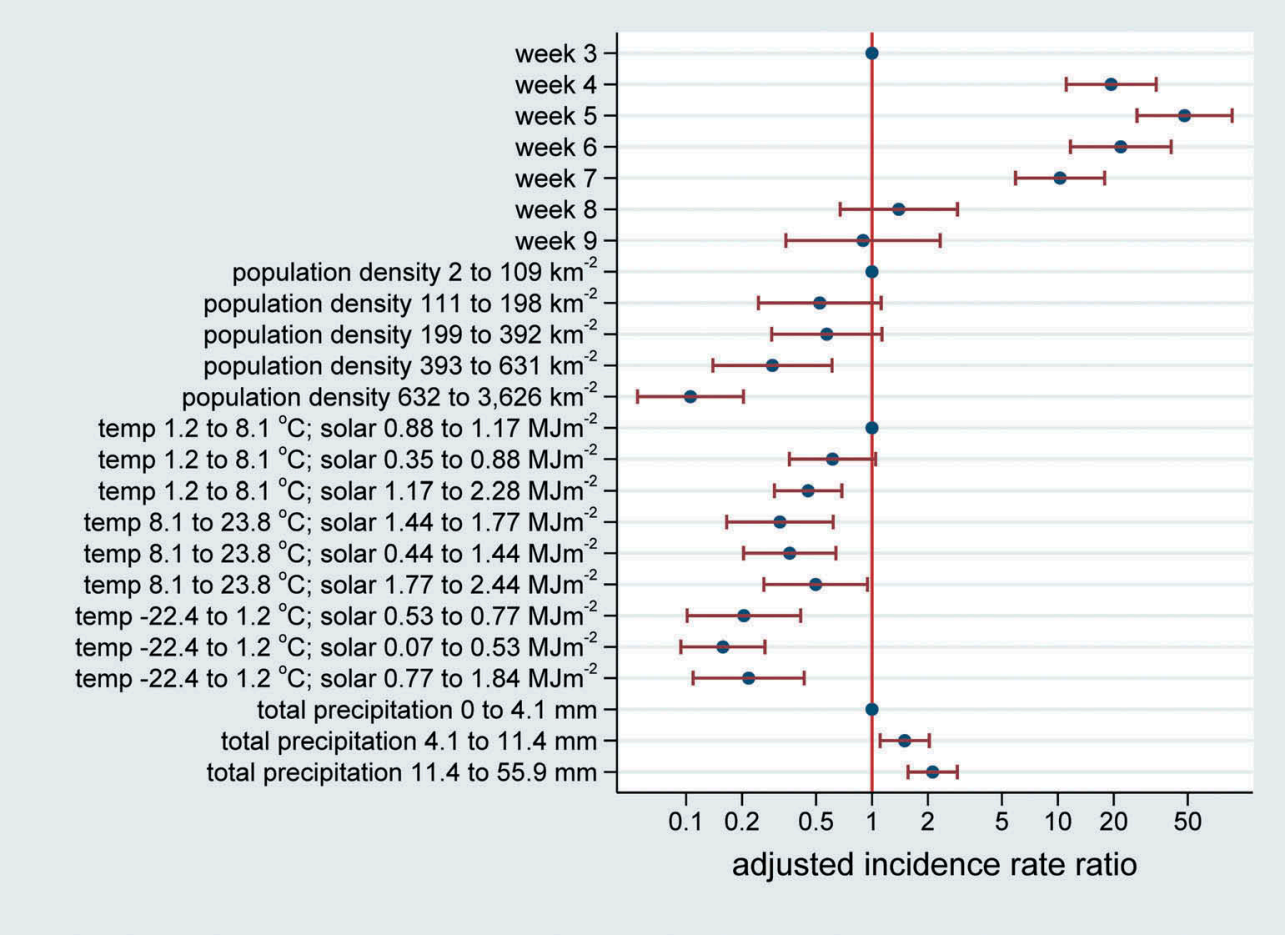
Adjusted incidence rate ratios with 95% confidence intervals in relation to weeks, population density, temperature, solar radiation and precipitation for a total of 18,069 COVID-19 cases in 559 0.25°grid cells, corresponding to a population of 151.2 million, in China (excluding Wuhan City) during January–February 2020

Figure 3 shows adjusted incidence rate ratios and 95% confidence intervals for the overall regression model. Adjusted incidence rate ratios (adjusted for week, population density and precipitation) for the composite temperature-radiation variable from the regression model are also shown in Figure 2. Most of the adjusted incidence rate ratios were significantly different from 1, evident in Figure 3 from the lack of overlap of 95% confidence intervals with the vertical line at an adjusted incidence rate ratio of 1.

The weather parameters for Wuhan City during the large epidemic peak there in January were average daily temperature 2.0°C, solar radiation 0.93 MJm^−2^ and precipitation 32 mm. This corresponds to the highest incidence rate ratio categories in the assessment for the rest of China shown in Figure 3.

## Discussion

These analyses clearly show variations in confirmed COVID-19 case incidence rates in China which are associated with weather during the week preceding case confirmation. An observational study of this kind cannot formally attribute cause and effect, and there remain uncertainties which have to be considered as unknown unknowns. Nevertheless, this assessment of the effects of weather on COVID-19 transmission in China suggests variations of a sufficient magnitude to have important possible consequences for understanding COVID-19 transmission in other settings. This assessment also illustrates the value of the detailed open-access data available both on confirmed COVID-19 cases and ecological parameters.

While the open-access individual confirmed case data is a hugely valuable resource, it is not able to tell the full story of the circumstances of each case. The nature of COVID-19 transmission is such that many cases will have no idea exactly where, when or how they acquired their infection, and so the location data for confirmed cases are inevitably more reflective of illness and treatment-seeking rather than infection. However, during the period of COVID-19 spread around China, following the initial epicentre epidemic in Wuhan City, the Chinese authorities implemented stringent infection reduction and travel restriction measures in many locations, the exact nature and chronology of which are not documented. The somewhat counterintuitive relationship seen in this assessment between population density and COVID-19 incidence very possibly reflects the effectiveness of infection control measures targeted at densely populated urban areas. However, records of cases in 559 locations outside Wuhan City, as shown in Figure 1(a), show that there was widespread nationwide transmission, even though only a single confirmed case was recorded in 20% of locations with cases. This possibly speaks to the effectiveness of control measures in many places, as well as the proportions of actual cases tested and confirmed.

Among the unknown unknowns, the effect of weather on human behaviour, and thus on behavioural risks for acquiring COVID-19, may also be important. The incidence of infections in China was markedly lower at very low temperatures, which might be related to characteristics of the virus, but equally may reflect reduced social contact when it is very cold outside. Conversely, brighter, drier weather may stimulate levels of social interaction, and thereby possibly counteract direct effects of heat and light on viruses. The complex observed relationship between temperature and solar radiation, as shown in Figure 2, is important, because the effects of temperature and light, particularly in the ultra-violet spectrum, have been shown to be associated with seasonal viral activity in other contexts [17]. In this assessment, the independent association of precipitation with COVID-19 incidence rates was also appreciable. A recent meteorological analysis showed very similar weather patterns across a number of COVID-19 hotspots, including Wuhan City, in a corridor 30° to 50° North in early 2020 [18]. This is congruent with findings here that weather conditions in Wuhan City during January corresponded to the highest risk categories as assessed across the rest of China.

Despite possible weaknesses around the case data, one of the strengths of this assessment is that all the other data were sourced from global data models that are totally independent of the COVID-19 data from China. Additionally, since China is a very large and geographically varied country, assessment of COVD-19 incidence was made over a very wide range of weather, as evident from Figure 1(c–f), and detailed daily weather data were sourced on a small-area basis at 0.25° resolution. Although there may have been local variations in the rigour with which COVID-19 cases in China were identified, tested and confirmed, it seems unlikely that such variations would have been systematically related to weather in previous weeks. In addition, measures representing intuitively accessible concepts in any location (warmth, sunshine and rainfall) were used, and allowed to compete in a multivariable model against COVID-19 incidence rate ratios, to produce findings that are directly understandable in public health terms. Generalisability of these findings beyond the Chinese context cannot be assumed, but there is no suggestion from these results that there was any combination of weather that would arrest COVID-19 transmission on a seasonal basis. An initial assessment of this kind, while not intended to be hypothesis-driven, is likely to generate hypotheses for further research. The associations demonstrated here between weather and transmission also raise an important specific research question. Recent progress in mitigating the COVID-19 pandemic is demonstrating the importance of social contact for transmission [19], and since social contact in many cultures also varies with weather conditions, it will be important to further investigate the weather – transmission – social contact complex in various settings.

## Conclusion

While this assessment showed appreciable associations between COVID-19 incidence rates and weather patterns in China during January–February 2020, this does not amount to establishing a clear cause and effect relationship. However, it does not support any generalisations to the effect that the COVID-19 pandemic will simply go away given some nice summer weather. The size of the associations between weather and incidence in China is very much of public health interest in understanding the continuing spread of the SARS-CoV-2 virus around the world, across different climate zones. The possibility that transmission is reduced during periods of brighter, warmer, drier weather, or increased during duller, cooler, wetter weather is important. Further assessments of this kind in other locations and seasons are needed to build a full picture, but meteorological data should be considered for inclusion in overall models of COVID-19 epidemiology and pandemic dynamics.

## Paper context

The availability of open-access individual-level case data from the COVID-19 epidemic in China during January–February 2020 provides an opportunity to assess disease transmission against local weather conditions at the time, available in global databases. This is potentially important information for other countries experiencing epidemics of COVID-19, allowing local weather conditions to be considered in estimates and forecasts of epidemic progression in other settings, and the prediction of possible resurgences during favourable transmission conditions.
